# How Does Obesity Cause Cancer?

**DOI:** 10.3390/cancers13215330

**Published:** 2021-10-23

**Authors:** Kyle Lee Hoehn

**Affiliations:** 1 School of Biotechnology and Biomolecular Sciences, University of New South Wales, Sydney, NSW 2052, Australia; k.hoehn@unsw.edu.au; 2 Department of Pharmacology, University of Virginia, Charlottesville, VA 22908, USA

This series comprises 14 articles (5 original articles and 9 reviews) that investigate connections between excess body mass and cancer risk or cancer treatment response. In one of the largest epidemiological studies in the United Kingdom, excess body mass was clearly associated with more than a dozen types of cancer, including a 41% increased risk of uterine cancer and a 10% or more risk of developing gallbladder, kidney, liver, and colon cancers, whereas being underweight decreased the risk of prostate and post-menopausal breast cancers [1]. Despite the correlation between excess body mass and cancer, a causal connection between excess weight and cancer remains to be definitively determined.

One example where correlation may not infer causation is a scenario where excess dietary sugar intake independently drives both obesity and cancer growth. My lab has been interested in this area of research following unexpected but reproducible results from well-controlled nutritional studies in mice, where dietary sugar intake was a far more powerful driver of liver tumourigenesis than obesity in the diethylnitrosamine model [2,3]. In this series, we called for manuscripts that investigated the cause versus correlation between obesity and cancer development.

The articles accepted into this series focus on liver [4,5,6], uterine [7], skin [8], breast [9,10,11], prostate [12], colon [13,14], pancreatic [15], and acute lymphoblastic leukemia cancers [16], with a general overview of the potential impacts of obesity-related changes in host metabolism on cancer development and/or progression [17].

For the liver articles, Smeuninx and colleagues highlight the complex relationship between fatty liver disease and liver cancer while discussing potential mechanisms of liver tumourigenesis through inflammation, oxidative stress, and the gut microbiome [5]. They detail the potential utility of drugs aimed at correcting fatty liver disease for liver cancer prevention and treatment. Rajesh and colleagues outline potential links between liver cancer and obesity-related changes in the gut microbiome, ER stress, oxidative stress and epigenetic changes during hepatocarcinogenesis with a strong focus on changes in signal transduction pathways and therapeutic interventions [6]. Huang J.C. and colleagues describe a relationship between the increased expression of dipeptidyl peptidase DPP9 in obese patients with liver cancer, where high expression is associated with poor survival, thus potentially implicating DPP9 in liver cancer pathogenesis [4].

Byrne and colleagues comprehensively review epidemiological data for endometrial cancer patients in the context of body mass index and glycaemia to highlight a recurring link between hyperglycaemia and cancer that can be independent of obesity, i.e., type 1 diabetics [7]. They also review data in cell and animal models that support a role for sugar in endometrial cancer growth and survival.

Smith and colleagues discuss in detail an obesity paradox in patients with cutaneous melanoma where increased adiposity is associated with improved therapeutic response and patient survival [8]. The relationship between obesity and improved patient response remains unclear but may have links to underlying effects of inflammation and immune response.

In the context of breast cancer, Cortesi and colleagues describe how clinically meaningful weight loss through lifestyle intervention could contribute to improving overall survival in obese patients compared to overweight subjects [9]. Huang J.Y. and colleagues detail evidence linking adipose-derived stem cells and the fat-derived hormone visfatin to breast cancer growth and metastasis [10]. Finally, Kolb and Zhang review the potential role of inflammation in linking obesity to estrogen-positive breast cancer in post-menopausal women [11].

The amount of adipose tissue that surrounds the prostate is associated with a more aggressive phenotype, but no studies have investigated prostate adipose biology in the context of cancer phenotypes. Here, Miladinovic and colleagues compare periprostatic adipose tissue with the subcutaneous adipose tissue of prostate cancer patients to identify unique features of prostate-associated adipose tissue compared to subcutaneous tissue. They identified differences across adipose depots but did not identify an association between excess body mass and peri-prostate adipose size, lipolysis, or lipid composition that could link to tumour aggressiveness [12].

Nam and colleagues investigated data archives for almost 10 million Korean adults over 8 years for the association between excess body mass or waist circumference and the risk of colorectal cancer [13]. They identified that increased waist circumference, but not body mass index, had a hazard ratio of 1.10 for the development of colorectal cancer. The authors suggest that central or visceral adiposity may be a more important risk factor for colorectal cancer in the Korean population than overall body mass. In a separate epidemiological study comparing the United States, China, and the world, Ye and colleagues review the associations with obesity and describe potential molecular mechanisms linked to nutrition, hormones, inflammation, and dysbiosis [14].

Brocco and colleagues review evidence linking excess adipose tissue to pancreatic cancer, including both local adipose tissue near the pancreas and distant tissue [15]. The effects of excess body mass and adiposity on stromal cell function, adipose inflammation, adipokine production, and gut microbiome are described in detail.

Excess body mass is linked to paediatric acute lymphoblastic leukemia (ALL), but little is known about the causal role of excess weight. In this edition, Dushnicky and colleagues review the potential effects of obesity on ALL risk in the context of endocrine, metabolic and immune dysregulation [16].

Finally, Wright and colleagues review the potential links between body mass, diet, and host metabolism to how well patients respond to anti-cancer treatment [17]. Together, these manuscripts help to us understand the complex relationship between excess body weight and cancer, and highlight idiosyncrasies in different types of cancer and model systems that may be useful in further dissecting the role of obesity and cancer incidence, progression, and/or sensitivity to treatment.

## References

[1] Bhaskaran K., Douglas I., Forbes H., Silva I.D.S., Leon D., Smeeth L. (2014). Body-mass index and risk of 22 specific cancers: A population-based cohort study of 5·24 million UK adults. Lancet.

[2] Healy M.E., Chow J.D.Y., Byrne F.L., Breen D.S., Leitinger N., Li C., Lackner C., Caldwell S.H., Hoehn K.L. (2015). Dietary effects on liver tumor burden in mice treated with the hepatocellular car-cinogen diethylnitrosamine. J. Hepatol..

[3] Healy M.E., Lahiri S., Hargett S.R., Chow J.D., Byrne F., Breen D.S., Kenwood B.M., Taddeo E.P., Lackner C., Caldwell S.H. (2016). Dietary sugar intake increases liver tumor incidence in female mice. Sci. Rep..

[4] Huang J., Emran A., Endaya J., McCaughan G., Gorrell M., Zhang H. (2021). DPP9: Comprehensive In Silico Analyses of Loss of Function Gene Variants and Associated Gene Expression Signatures in Human Hepatocellular Carcinoma. Cancers.

[5] Smeuninx B., Boslem E., Febbraio M.A. (2020). Current and Future Treatments in the Fight Against Non-Alcoholic Fatty Liver Disease. Cancers.

[6] Rajesh Y., Sarkar D. (2020). Molecular Mechanisms Regulating Obesity-Associated Hepatocellular Car-cinoma. Cancers.

[7] Byrne F.L., Martin A.R., Kosasih M., Caruana B.T., Farrell R. (2020). The Role of Hyperglycemia in Endometrial Cancer Pathogenesis. Cancers.

[8] Smith L.K., Arabi S., Lelliott E.J., McArthur G.A., Sheppard K.E. (2020). Obesity and the Impact on Cutaneous Melanoma: Friend or Foe?. Cancers.

[9] Cortesi L., Sebastiani F., Iannone A., Marcheselli L., Venturelli M., Piombino C., Toss A., Federico M. (2020). Lifestyle Intervention on Body Weight and Physical Activity in Patients with Breast Cancer can reduce the Risk of Death in Obese Women: The EMILI Study. Cancers.

[10] Huang J.-Y., Wang Y.-Y., Lo S., Tseng L.-M., Chen D.-R., Wu Y.-C., Hou M.-F., Yuan S.-S.F. (2020). Visfatin Mediates Malignant Behaviors through Adipose-Derived Stem Cells In-termediary in Breast Cancer. Cancers.

[11] Kolb R., Zhang W. (2020). Obesity and Breast Cancer: A Case of Inflamed Adipose Tissue. Cancers.

[12] Miladinovic D., Cusick T., Mahon K.L., Haynes A.-M., Cortie C.H., Meyer B.J., Stricker P.D., Wittert G.A., Butler L.M., Horvath L.G. (2020). Assessment of Periprostatic and Subcutaneous Adipose Tissue Lipolysis and Adipocyte Size from Men with Localized Prostate Cancer. Cancers.

[13] Nam G.E., Baek S.-J., Choi H.B., Han K., Kwak J.-M., Kim J., Kim S.-H. (2020). Association between Abdominal Obesity and Incident Colorectal Cancer: A Na-tionwide Cohort Study in Korea. Cancers.

[14] Ye P., Xi Y., Huang Z., Xu P. (2020). Linking Obesity with Colorectal Cancer: Epidemiology and Mechanistic Insights. Cancers.

[15] Brocco D., Florio R., De Lellis L., Veschi S., Grassadonia A., Tinari N., Cama A. (2020). The Role of Dysfunctional Adipose Tissue in Pancreatic Cancer: A Molecular Per-spective. Cancers.

[16] Dushnicky M.J., Nazarali S., Mir A., Portwine C., Samaan M.C. (2020). Is There A Causal Relationship between Childhood Obesity and Acute Lym-phoblastic Leukemia? A Review. Cancers.

[17] Wright C.M., Shastri A.A., Bongiorno E., Palagani A., Rodeck U., Simone N.L. (2020). Is Host Metabolism the Missing Link to Improving Cancer Outcomes?. Cancers.

